# The efficacy and safety of acetylcysteine combined with budesonide nebulization in treating mycoplasma pneumonia in children: a meta-analysis

**DOI:** 10.3389/fped.2025.1574257

**Published:** 2025-06-20

**Authors:** Yinan He, Rongmei Huang

**Affiliations:** ^1^ Pediatric Intensive Care Unit, Ya'an People’s Hospital, Ya'an, China; ^2^ Department of Obstetrics and Gynecology, Ya'an People’s Hospital, Ya'an, China

**Keywords:** mycoplasma pneumonia, children, acetylcysteine, budesonide, curative effect, meta-analysis

## Abstract

**Background:**

The aim of this meta-analysis was to systematically evaluate the clinical efficacy and safety of acetylcysteine combined with budesonide nebulization in treating Mycoplasma pneumonia in children.

**Methods:**

We systematically searched eight electronic databases for randomized controlled trials (RCTs) evaluating the use of acetylcysteine combined with budesonide nebulization in treating Mycoplasma pneumonia in children, from database inception through December 2024, and performed data analysis using a random-effects model.

**Results:**

The 29 RCTs involving 4,300 children were conducted. The experimental group received acetylcysteine plus budesonide treatment, while the comparison group received budesonide alone. Results showed the experimental group had a significantly higher overall clinical efficacy rate (RR = 1.16, 95% CI = 1.13–1.20, *I*^2^ = 16%). The experimental group also had a significantly lower incidence of diarrhea (RR = 0.17, 95% CI = 0.05–0.54, *I*^2^ = 0%), with no significant difference in other adverse events. The experimental group had significantly shorter times to resolution of cough (SMD = −2.11, 95% CI = −2.65 to −1.57, *I*^2^ = 97%), moist rale (SMD = −1.91, 95% CI = −2.50 to −1.33, *I*^2^ = 97%), and fever (SMD = −1.70, 95% CI = −2.26 to −1.14, *I*^2^ = 95%). Post-treatment, the experimental group had significantly lower C-reactive protein levels (SMD = −1.44, 95% CI = −1.92 to −0.97, *I*^2^ = 91%).

**Conclusion:**

Compared with budesonide monotherapy, acetylcysteine combined with budesonide significantly improved the clinical efficacy in children with Mycoplasma pneumonia while not increasing the risk of adverse events, and represents a safe and effective treatment option.

## 1 Introduction

Pneumonia is a common respiratory disease that poses a severe threat to the health of children and is one of the leading causes of morbidity and mortality from infectious diseases in children under 5 years old. Annually, approximately 1.6–1.9 million children under five die from pneumonia, accounting for nearly one-fifth of all deaths globally in this age group (1). In developing countries or regions, this burden is even more severe, with approximately 1.56 million new cases occurring annually (2). In China, the incidence of pneumonia among children is significantly higher than that in high-income countries: the urban incidence rate is 65.8 per 1,000 person-years for children under 5 years old (compared to 44.6 in high-income countries), 17.37 for those aged 5–9 years, and 3.07 for those aged 10–17 years (3, 4), with a case fatality rate ranging from 0.32‰ to 1.09‰. This accounts for 8% of all-cause mortality, making it the leading cause of infection-related deaths in children under five (5, 6).

Nebulized inhalation is a method of drug delivery that directly targets the respiratory tract and lungs. It offers advantages such as rapid onset of action, high local drug concentration, convenience of use, and relatively minimal systemic adverse reactions, making it a key modality in the treatment of respiratory diseases (7). At present, nebulized inhalation formulations have achieved substantial progress in China, with more than ten formulations based on active ingredients currently available on the market (8). Among them, acetylcysteine is an expectorant. The thiol group in its molecular structure can break the disulfide bonds between mucin molecules, directly dissolve and liquefy mucus for clearance. Additionally, it improves ciliary motion, increases alveolar surfactant production, eliminates oxygen free radicals, and disrupts bacterial biofilms (9, 10). Budesonide is one of the most potent inhaled corticosteroid inhaled corticosteroid (ICS) agents with local anti-inflammatory effects in the airway. It inhibits airway inflammation through multiple mechanisms, reducing airway hyperresponsiveness and bronchospasm (11). Budesonide is the only ICS recommended in the World Health Organization (WHO) Model List of Essential Medicines for Children for asthma management. It is also classified as Category B for pregnancy safety by the Food and Drug Administration (FDA). Budesonide is approved for nasal and inhalation formulations and is currently the only nebulized ICS approved for use in children aged 4 years or younger (12, 13).

Acetylcysteine and budesonide are both guideline-recommended treatments for Mycoplasma pneumonia in children. However, evidence-based clinical data on their combined use are currently limited. Therefore, we conducted this meta-analysis to systematically evaluate the clinical efficacy and safety of the use of nebulized acetylcysteine combined with budesonide in treating Mycoplasma pneumonia in children.

## 2 Methods

We reported the meta-analysis according to the Preferred Reporting Items for Systematic Reviews and Meta-Analyses (PRISMA 2020) statement for systematic reviews of interventions (14). It is registered with the International Prospective Register of Systematic Reviews (PROSPERO registration number: CRD42022320354). Given that this is a review study, we were not required to secure approval for the study protocol from an ethics committee or institutional review board, nor was informed consent from study participants necessary.

### 2.1 Inclusion and exclusion criteria

The inclusion and exclusion criteria were established in advance. Randomized controlled trials (RCTs) evaluating the efficacy of nebulized acetylcysteine combined with budesonide for treating Mycoplasma pneumonia in children were searched. The specific inclusion and exclusion criteria are as follows:

#### 2.1.1 Inclusion criteria

(1) Participants: Children diagnosed with pediatric bronchopneumonia according to the criteria in ZHU FUTANG PRACTICE OF PEDIATRICS; parents of the children willing to cooperate with observers and provide informed consent before treatment; age range: 2–11 years; no other severe respiratory diseases; no treatment with corticosteroids or antibiotics in the past month. (2) Interventions: Children receiving nebulized acetylcysteine combined with budesonide therapy. (3) Comparison: Children receiving nebulized budesonide therapy. (4) Outcomes: The included outcomes consisted of at least one of the following: overall clinical efficacy rate, duration to resolution of clinical symptoms (cough, fever, and pulmonary moist rales), C-reactive protein levels, or adverse events. (5) Study design: RCTs.

#### 2.1.2 Exclusion criteria

(1) Reviews, case reports, conference abstracts, and other non-controlled studies. (2) Studies where either the treatment or comparison group involved additional medications. (3) Studies with incomplete data or data that could not be extracted effectively. (4) Duplicate publications.

### 2.2 Search strategy

We conducted an electronic database search in the Cochrane Library, PubMed, Embase, Web of Science, Chinese Biology Medicine Disc, China National Knowledge Infrastructure, Wanfang Data, and the China Science and Technology Journal Database (VIP). There were no restrictions on date or language. The last search was updated in December 2024. The search strategy was as follows: ((((“Child”[Mesh]) OR (Child OR Children OR Pediatric)) AND (((“Pneumonia”[Mesh]) OR “Pneumonia, Mycoplasma”[Mesh]) OR (Pneumonia OR “Pneumonia, Mycoplasma” OR “Mycoplasma pneumonia”))) AND ((“Budesonide”[Mesh]) OR (Budesonide))) AND ((“Acetylcysteine”[Mesh]) OR (Acetylcysteine OR N-acetylcysteine)). To minimize bias, we searched for ongoing registered clinical trials and unpublished papers. In addition, we manually searched the references of relevant studies to maximize the retrieval of relevant studies. The above search procedures were independently performed by two researchers (Y.H. and R.H.), which were then cross-checked.

### 2.3 Study selection and data extraction

The two researchers (Y.H. and R.H.) performed an initial screening of studies by reading titles and abstracts based on the inclusion and exclusion criteria, followed by a full-text review to determine the studies ultimately included in this meta-analysis. Two researchers (Y.H. and R.H.) independently extracted the following data: first author, publication year, country of study, study design, sample size, patient age, intervention measures for the experimental and comparison groups, disease course, treatment duration, and outcomes. In cases of disagreement during the process, the two researchers resolved the discrepancies through discussion or sought arbitration from a third researcher.

### 2.4 Assessment of risk of bias in included studies

The two researchers (Y.H. and R.H.) independently conducted risk of bias (RoB) assessments in seven domains using the Cochrane Collaboration's risk of bias tool: random sequence generation, allocation concealment, blinding of participants and personnel, blinding of outcome assessment, incomplete outcome data, selective reporting, and other sources of bias. Each domain was rated as “low risk,” “high risk,” or “unclear risk.”

### 2.5 Data synthesis and analysis

We used Review Manager (version 5.4, Cochrane Collaboration, Oxford, UK) software to perform the meta-analysis. A Mantel-Haenszel random-effects model was used to compute pooled effect estimates for primary and secondary outcomes. We pooled dichotomous outcomes using relative risk (RR) and the 95% confidence intervals (CI), and continuous outcomes using standardized mean difference (SMD) and the 95% CI. Publication bias was evaluated through the construction of a funnel plot and the application of Egger's test.

The overall clinical efficacy rate and adverse events (total incidence, nausea and vomiting, throat discomfort, dizziness, diarrhea, and rash) were defined as the primary outcomes. Secondary outcomes included the time to resolution of clinical symptoms (cough, fever, and pulmonary moist rale) and C-reactive protein levels.

The criteria for evaluating clinical efficacy were as follows: (1) Marked efficacy: The patient's body temperature returned to normal, and symptoms such as cough, headache, throat pain, and pulmonary rales disappeared completely; chest x-rays showed no lung shadows. (2) Effective: The patient's body temperature returned to normal range, with occasional occurrences of symptoms such as cough, headache, and throat pain; pulmonary rales were almost resolved, and chest x-rays showed significant reduction in lung shadows. (3) Ineffective: No improvement or worsening of the above signs and symptoms. The overall clinical efficacy rate was calculated as follows: (Marked efficacy + Effective cases)/Total cases × 100%.

### 2.6 Subgroup and the sensitivity analysis

We intend to perform subgroup and sensitivity analyses to evaluate the robustness of key findings regarding the overall clinical efficacy rate and the total incidence of adverse events. Subgroup analyses will be conducted based on the risk of bias assessment of the included studies. Studies classified as having “unclear” or “high risk” in terms of blinding or allocation concealment will be designated as the high-risk group, while those with lower risk will be categorized as the low-risk group. This approach aims to evaluate the influence of bias risk in the included studies on the conclusions of the meta-analysis.

## 3 Results

### 3.1 Results of literature retrieval

**Figure 1:**
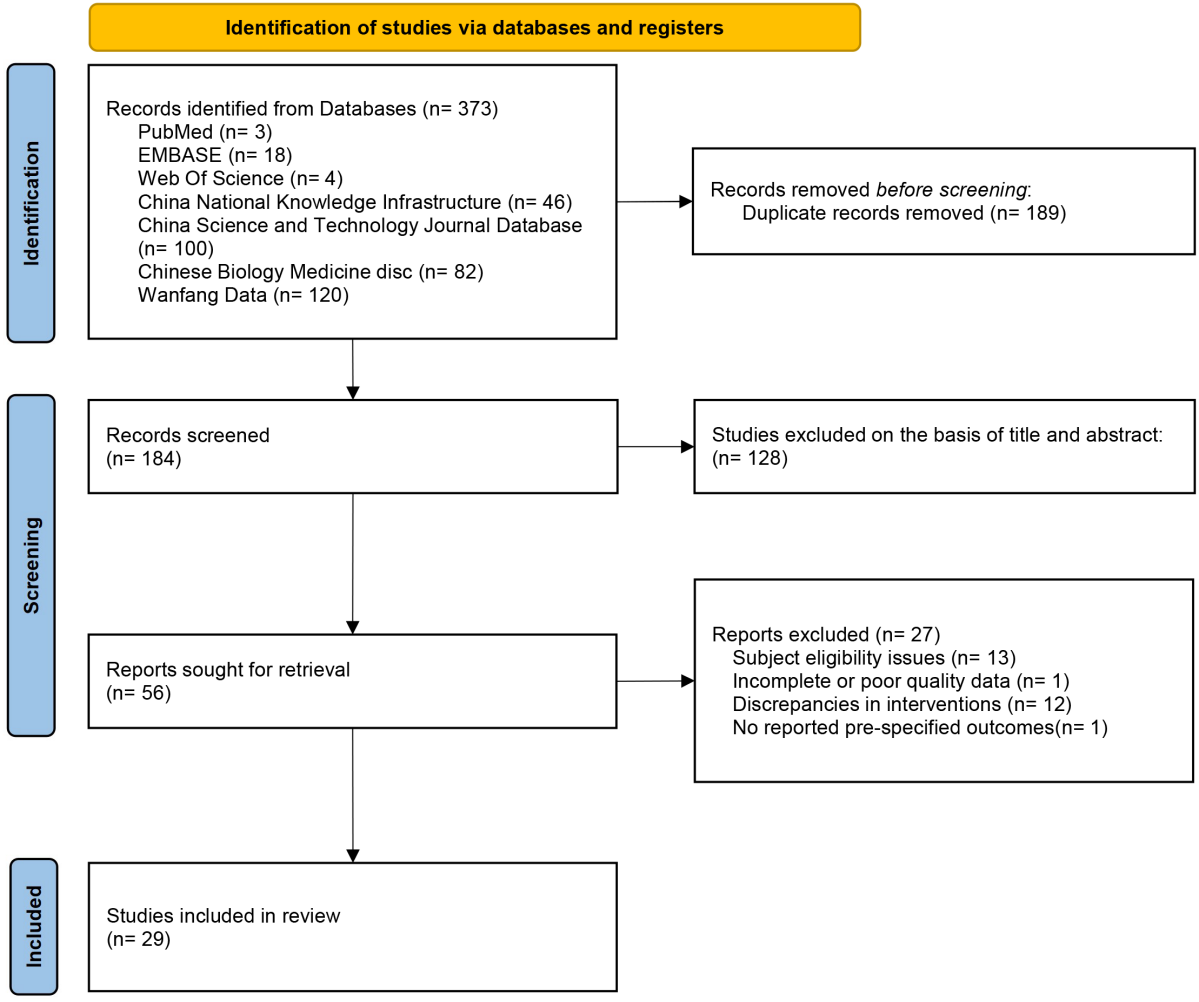
Flowchart of the screening process.

The Figure 1 illustrates the PRISMA flowchart of the study selection process. The search strategy identified 373 studies. After removing duplicates, 184 studies remained. Of these, 128 studies were excluded after screening titles and abstracts, and 27 studies were excluded after full-text review (13 studies had inappropriate subjects; 1 study had incomplete data; 12 studies had inappropriate interventions; 1 study did not report predefined outcomes). A total of 29 RCTs (15–43) were included.

### 3.2 General characteristics of included studies

The Table 1 summarizes the baseline characteristics of the 29 RCTs. All trials were investigator-initiated, post-marketing studies of acetylcysteine and budesonide, entirely conducted in China, and all were RCTs. The trials were conducted between 2017 and 2024, encompassing 4,300 children, with 2,155 in the experimental group and 2,145 in the control group. The patients were aged 2–11 years. The experimental group received nebulized acetylcysteine combined with budesonide, while the control group received nebulized budesonide alone, for a duration of 7–14 days.

**Table 1:** Basic characteristics of included trials and subjects.

Study	Year	Country	Study type	Treatments		No. of patients E/C	Age (M ± SD or median, years) E/C	Disease course (M ± SD or median, day)	Treatment duration	Outcomes
				E	C					
Chen, J., et al. (15)	2023	China	RCT	Acetylcysteine + Budesonide	Budesonide	60/60	7.27 ± 2.47/7.02 ± 2.58	4.97 ± 1.21/5.17 ± 1.29	14 D	①④
Cheng, J. (16)	2024	China	RCT	Acetylcysteine + Budesonide	Budesonide	30/30	4.34 ± 1.25/4.36 ± 1.45	9.23 ± 2.17/9.22 ± 2.27	14 D	①③④
Ci, M.M., et al. (17)	2023	China	RCT	Acetylcysteine + Budesonide	Budesonide	150/150	5.64 ± 2.01/6.71 ± 2.12	7.15 ± 1.86/7.03 ± 1.75	7 D	①
Gao, M.Q. (18)	2023	China	RCT	Acetylcysteine + Budesonide	Budesonide	100/100	2.86 ± 0.74/2.83 ± 0.77	3.83 ± 1.17/4.09 ± 1.21	7 D	①②④
Guo, H.F. (19)	2022	China	RCT	Acetylcysteine + Budesonide	Budesonide	40/40	3.08 ± 1.02/3.10 ± 0.95	5.03 ± 1.06/5.10 ± 1.11	7 D	①②③④
He, W.W. (20)	2020	China	RCT	Acetylcysteine + Budesonide	Budesonide	60/60	3.08 ± 1.25/3.01 ± 1.47	6.12 ± 1.20/6.53 ± 1.14	7 D	①④
Jia, J.W. (21)	2023	China	RCT	Acetylcysteine + Budesonide	Budesonide	38/38	3.22 ± 0.63/3.19 ± 0.66	6.84 ± 1.02/6.79 ± 1.13	7 D	①③④
Kong, M.Y., et al. (22)	2022	China	RCT	Acetylcysteine + Budesonide	Budesonide	52/52	5.92 ± 0.83/6.11 ± 0.79	4.71 ± 0.87/4.97 ± 0.96	7 D	①③
Li, P. (23)	2022	China	RCT	Acetylcysteine + Budesonide	Budesonide	50/50	4.33 ± 1.22/4.23 ± 1.22	9.21 ± 2.11/9.23 ± 2.13	7 D	①③④
Lin, Y.M. (24)	2019	China	RCT	Acetylcysteine + Budesonide	Budesonide	35/35	6.78 ± 2.65/6.29 ± 2.65	NA	7 D	①②④
Liu, H.F., et al. (25)	2023	China	RCT	Acetylcysteine + Budesonide	Budesonide	43/43	5.6 ± 1.0/6.0 ± 1.3	5.3 ± 0.6/5.9 ± 0.5	7 D	①③
Liu, M., et al. (26)	2023	China	RCT	Acetylcysteine + Budesonide	Budesonide	54/54	8.86 ± 2.05/8.92 ± 2.12	2.59 ± 0.83/2.60 ± 0.77	7 D	①②③④
Liu, W.H., et al. (27)	2023	China	RCT	Acetylcysteine + Budesonide	Budesonide	41/41	3.62 ± 1.13/3.45 ± 1.03	8.92 ± 2.68/8.77 ± 2.63	7 D	①④
Liu, Y., et al. (28)	2019	China	RCT	Acetylcysteine + Budesonide	Budesonide	450/450	2.5/2.3	NA	7 D	①②④
Ma, L., et al. (29)	2022	China	RCT	Acetylcysteine + Budesonide	Budesonide	45/45	2.91 ± 1.32/3.09 ± 1.40	8.67 ± 1.98/8.96 ± 1.91	7 D	①②④
Ma, X.L. (30)	2024	China	RCT	Acetylcysteine + Budesonide	Budesonide	40/40	4.12 ± 0.78/4.08 ± 0.77	10.02 ± 1.75/9.89 ± 1.81	7 D	①④
Song, H.L. (31)	2017	China	RCT	Acetylcysteine + Budesonide	Budesonide	60/60	1.5 ± 0.5/1.5 ± 0.6	5.5/5.5	7 D	①②④
Sun, D.Q., et al. (32)	2022	China	RCT	Acetylcysteine + Budesonide	Budesonide	50/50	6.60 ± 0.64/6.53 ± 0.61	5.11 ± 0.50/5.04 ± 0.48	7 D	①②
Tang, X.Y. (33)	2023	China	RCT	Acetylcysteine + Budesonide	Budesonide	38/38	3.92 ± 0.85/3.56 ± 0.78	4.95 ± 1.15/5.23 ± 1.12	7 D	①④
Wang, F. (34)	2020	China	RCT	Acetylcysteine + Budesonide	Budesonide	50/50	2.45 ± 0.78/2.34 ± 0.83	NA	NA	①②
Wang, G.Y. (35)	2023	China	RCT	Acetylcysteine + Budesonide	Budesonide	100/100	6.87 ± 0.54/6.82 ± 0.53	5.14 ± 1.12/5.12 ± 1.14	14 D	①②
Wang, J.S., et al. (36)	2021	China	RCT	Acetylcysteine + Budesonide	Budesonide	35/31	3.86 ± 0.86/3.95 ± 0.83	NA	7 D	①②③④
Wang, Y., et al. (37)	2023	China	RCT	Acetylcysteine + Budesonide	Budesonide	30/24	5.18 ± 2.33/5.70 ± 2.25	NA	14 D	①④
Xiao, F., et al. (38)	2022	China	RCT	Acetylcysteine + Budesonide	Budesonide	65/65	2.06 ± 0.43/2.05 ± 0.41	3.35 ± 0.91/3.38 ± 0.89	7 D	①②③④
Xiao, J.X., et al. (39)	2021	China	RCT	Acetylcysteine + Budesonide	Budesonide	185/185	6.13 ± 2.41/6.44 ± 2.56	6.40 ± 1.75/6.33 ± 1.58	7 D	①②④
Zhang, M., et al. (40)	2023	China	RCT	Acetylcysteine + Budesonide	Budesonide	100/100	5.09 ± 1.25/5.10 ± 1.32	5.79 ± 1.37/5.84 ± 1.41	7 D	①②
Zhang, Q.Q. (41)	2022	China	RCT	Acetylcysteine + Budesonide	Budesonide	44/44	3.26 ± 0.55/3.25 ± 0.52	6.27 ± 1.28/6.25 ± 1.25	7 D	①③
Zhao, Q.P. (42)	2024	China	RCT	Acetylcysteine + Budesonide	Budesonide	60/60	8.92 ± 3.67/8.36 ± 3.96	5.85 ± 0.68/5.22 ± 0.56	7 D	②③
Zhong, L.H., et al. (43)	2021	China	RCT	Acetylcysteine + Budesonide	Budesonide	50/50	2.14 ± 0.35/2.20 ± 0.38	3.48 ± 1.05/3.52 ± 1.08	7 D	①②④

E, experimental group; C, control group; No., number; M, mean; SD, standard deviation; NA, not available; D, day; ①, overall efficacy rate; ②, clinical symptoms (cough, lung moist rales, fever) subsided time; ③, C-reactive protein; ④, adverse events.

### 3.3 Risk-of-bias assessment

**Figure 2:**
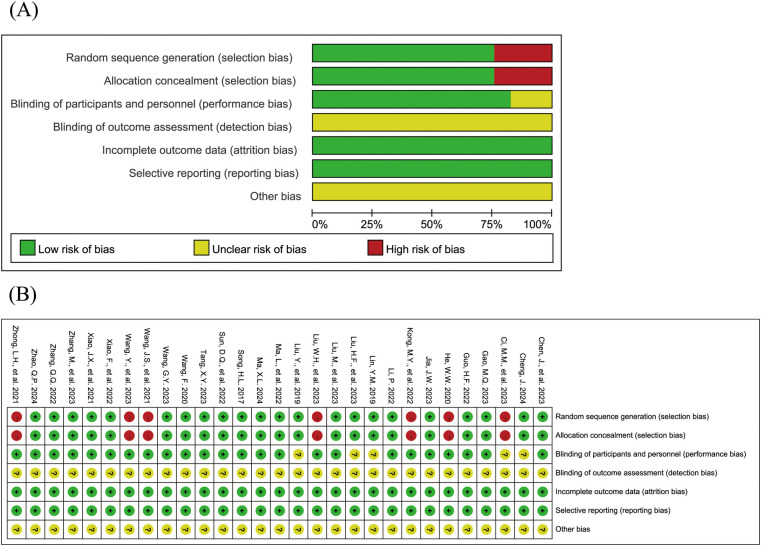
Risk of bias assessment results for the included studies (**A**: risk of bias graph; **B**: risk of bias summary).

The Figure 2 shows the risk of bias assessment results for the included studies. 22 studies (15, 16, 18, 19, 21, 23–26, 28–35, 38–42) reported both random sequence generation and allocation concealment; 1 study (15) was a double-blind trial, and 23 studies (18–23, 26, 27, 29–43) reported that patients provided informed consent. All studies had complete data with no selective reporting. None of the studies explicitly stated blinding of outcome assessment or other sources of bias.

### 3.4 Primary outcomes

#### 3.4.1 Overall clinical efficacy rate

**Figure 3:**
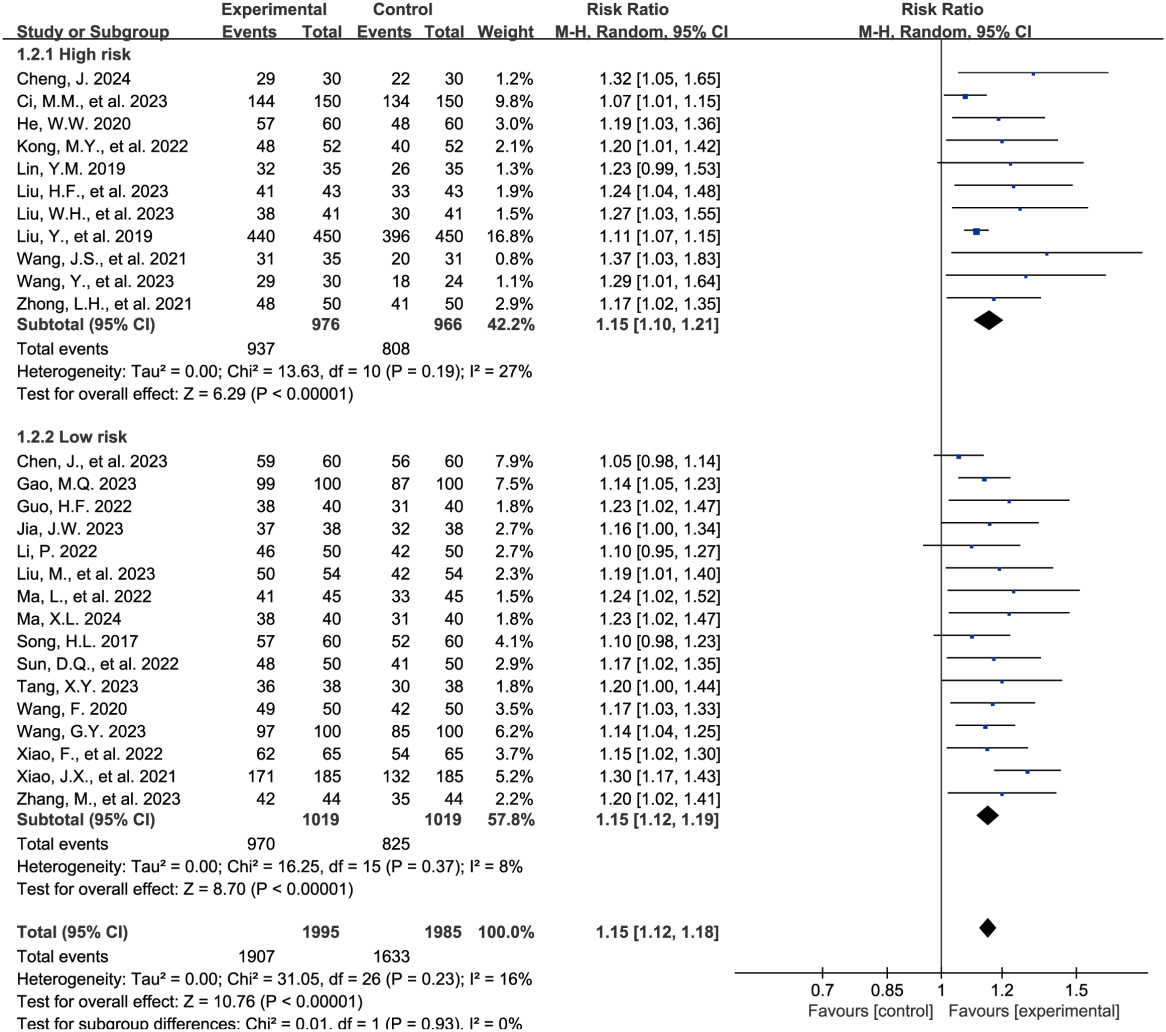
Forest plot of overall clinical efficacy rate.

The Figure 3 shows the forest plot for the overall clinical efficacy rate. 27 studies (15–40, 43) reported outcomes related to the overall clinical efficacy rate, encompassing 3,990 children, with 1,995 in the experimental group and 1,995 in the control group. Compared with the control group, the combination of acetylcysteine and budesonide resulted in a 15% increase in the overall clinical efficacy rate, with the difference being statistically significant (RR = 1.15, 95% CI = 1.12–1.18, *I*^2^ = 16%).

Subgroup analyses were conducted based on the risk of bias assessment. 11 studies (16, 17, 20, 22, 24, 25, 27, 28, 36, 37, 43) were categorized as the high-risk group due to elevated bias risk, while 16 studies (15, 18, 19, 21, 23, 26, 29–35, 38–40) were designated as the low-risk group. The findings indicated that, in both subgroups, the combination of acetylcysteine and budesonide significantly enhanced the overall clinical efficacy rate (high-risk group: RR = 1.15, 95% CI = 1.10–1.21, *I*^2^ = 27%; low-risk group: RR = 1.15, 95% CI = 1.12–1.19, *I*^2^ = 8%).

The Supplementary Figure S1 presents the funnel plot for the overall clinical efficacy rate. Analysis of the funnel plot reveals evidence of asymmetry, supported by Egger's test (*P* < 0.001), indicating potential publication bias in the pooled results.

#### 3.4.2 Adverse events

**Figure 4:**
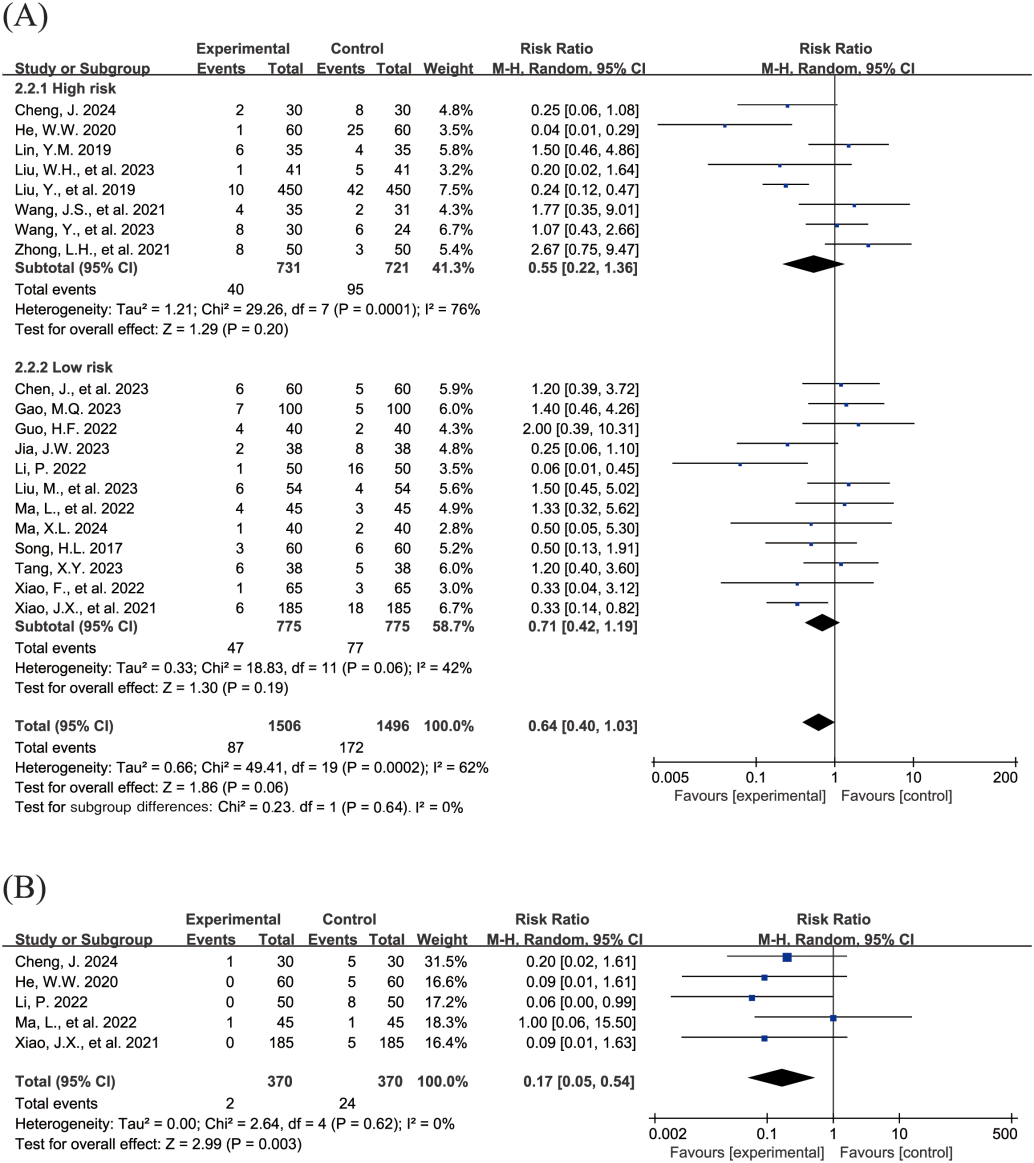
Forest plot of the incidence of adverse events (**A**: overall incidence of adverse events; **B**: incidence of diarrhea; **C**: incidence of nausea and vomiting; **D**: incidence of pharyngeal discomfort; **E**: incidence of dizziness; **F**: incidence of rash).

The Figure 4 shows the forest plot of the incidence of adverse events. 20 studies (15, 16, 18–21, 23, 24, 26–31, 33, 36–39, 43) reported the overall incidence of adverse events, involving 3,002 children, with 1,506 in the experimental group and 1,496 in the control group. Compared with the control group, children treated with acetylcysteine combined with budesonide experienced a 36% reduction in the overall incidence of adverse events; however, the result was not statistically significant (RR = 0.64, 95% CI = 0.40–1.03, *I*^2^ = 62%), as shown in Figure 4A. Specifically, the incidence of diarrhea in the experimental group was significantly lower than that in the control group (RR = 0.17, 95% CI = 0.05–0.54, *I*^2^ = 0%), as shown in Figure 4B. The incidence rates of other adverse events, including nausea and vomiting, pharyngeal discomfort, dizziness, and rash, did not show statistically significant differences. The specific results were as follows: nausea and vomiting (RR = 0.77, 95% CI = 0.44–1.34, *I*^2^ = 13%), as shown in Figure 4C; pharyngeal discomfort (RR = 1.52, 95% CI = 0.62–3.72, *I*^2^ = 0%), as shown in Figure 4D; dizziness (RR = 0.84, 95% CI = 0.29–2.47, *I*^2^ = 0%), as shown in Figure 4E; and rash (RR = 1.24, 95% CI = 0.31–5.00, *I*^2^ = 0%), as shown in Figure 4F.

**Figure:**
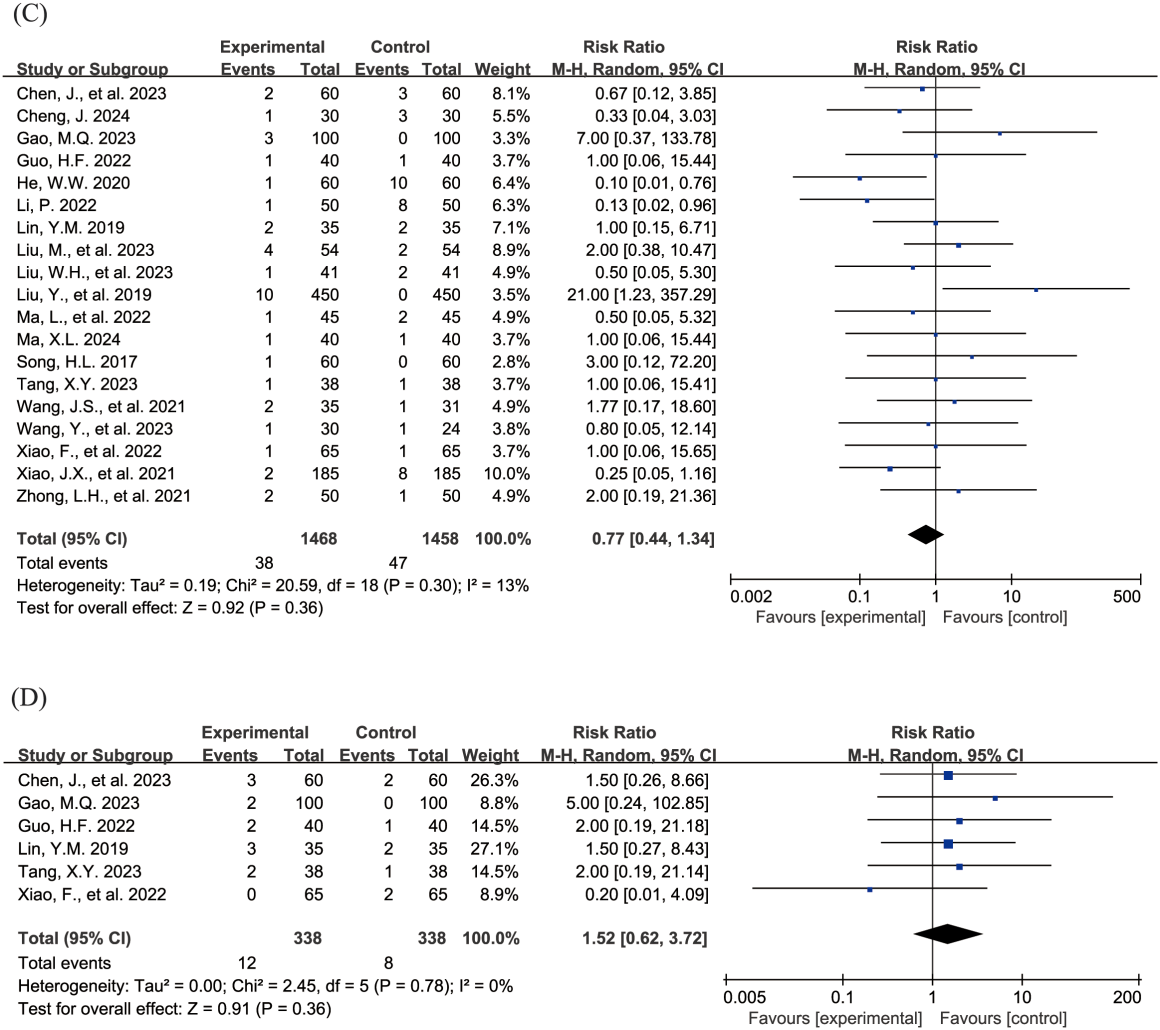


**Figure:**
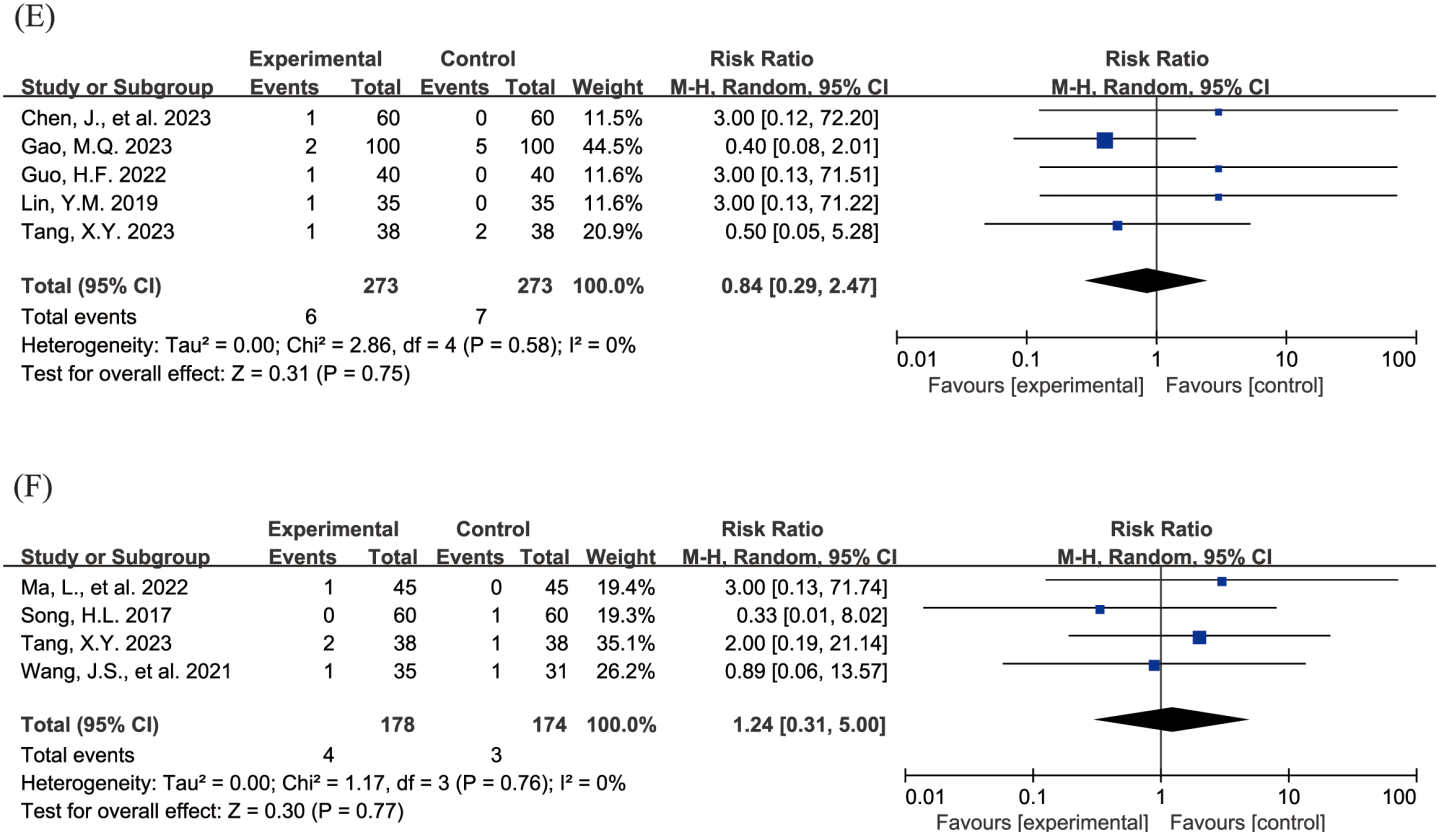


Subgroup analyses were conducted to evaluate the total incidence of adverse events, with the high-risk group comprising 8 studies (16, 20, 24, 27, 28, 36, 37, 43) and the low-risk group encompassing 12 studies (15, 18, 19, 21, 23, 26, 29–31, 33, 38, 39). The findings indicated that, in both subgroups, the differences in the total incidence of adverse events were not statistically significant (high-risk group: RR = 0.55, 95% CI = 0.22–1.36, *I*^2^ = 76%; low-risk group: RR = 0.71, 95% CI = 0.42–1.19, *I*^2^ = 42%).

The Supplementary Figure S2 presents the funnel plot for the total incidence of adverse events. The funnel plot demonstrates satisfactory symmetry, supported by Egger's test (*P* = 0.891, >0.05), indicating that publication bias is not significant in the pooled results.

### 3.5 Secondary outcomes

#### 3.5.1 Resolution time of clinical symptoms (cough, pulmonary moist rale, and fever)

**Figure 5:**
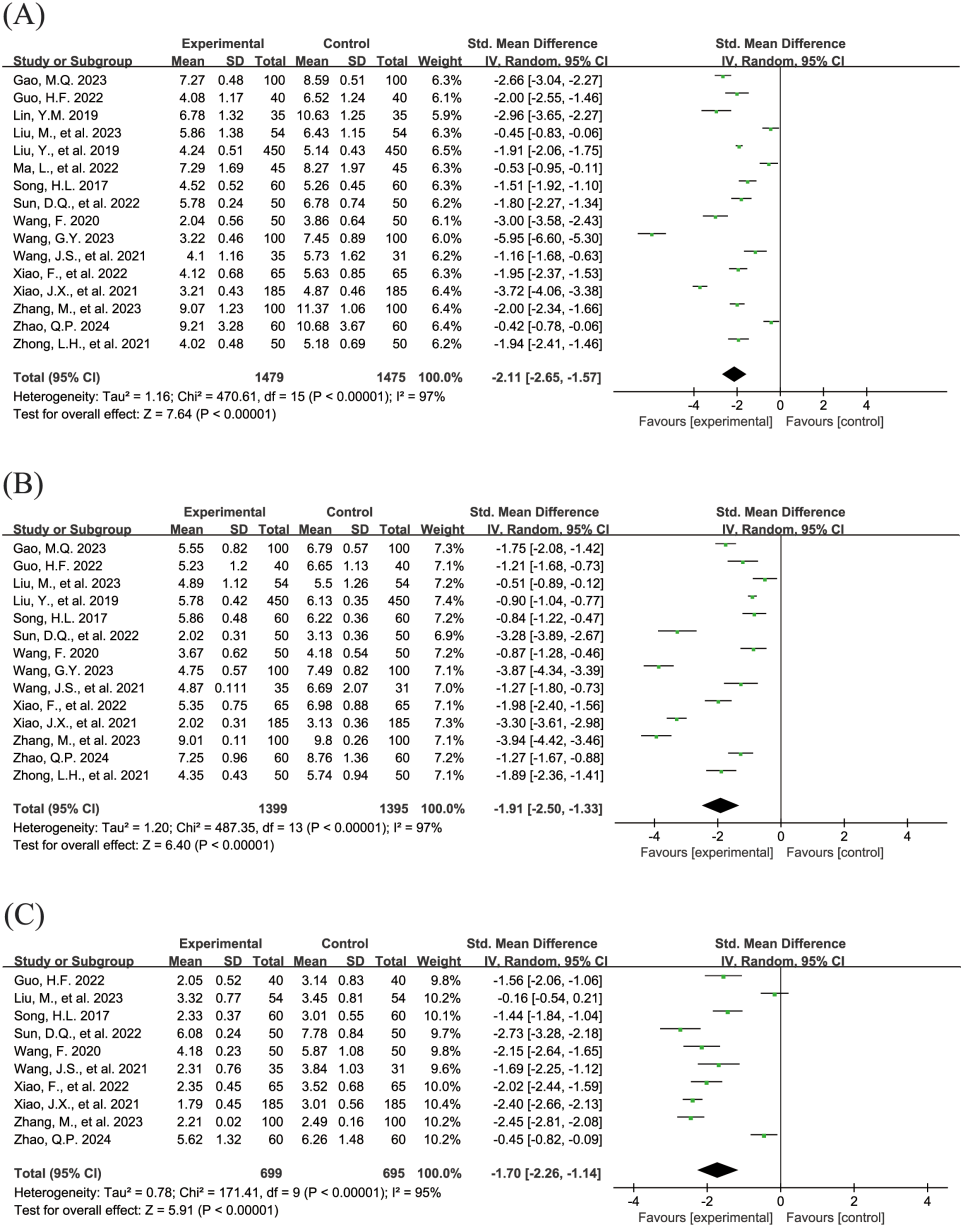
Forest plot of the resolution time of clinical symptoms. **(A)** Resolution time of cough. **(B)** Resolution time of pulmonary moist rales. **(C)** Resolution time of fever.

The Figure 5 shows the forest plot of the resolution time of clinical symptoms. In this meta-analysis, 16 studies (18, 19, 24, 26, 28, 29, 31, 32, 34–36, 38–40, 42, 43) reported the resolution time of cough (SMD = −2.11, 95% CI = −2.65 to −1.57, *I*^2^ = 97%), as shown in Figure 5A. 14 studies (18, 19, 26, 28, 31, 32, 34–36, 38–40, 42, 43) reported the resolution time of pulmonary moist rale (SMD = −1.91, 95% CI = −2.50 to −1.33, *I*^2^ = 97%), as shown in Figure 5B. 10 studies (19, 26, 31, 32, 34, 36, 38–40, 42) reported the resolution time of fever (SMD = −1.70, 95% CI = −2.26 to −1.14, *I*^2^ = 95%), as shown in Figure 5C. All pooled results showed that the resolution time of clinical symptoms in the experimental group was shorter than that in the control group, and the differences were statistically significant.

#### 3.5.2 C-reactive protein levels

**Figure 6:**
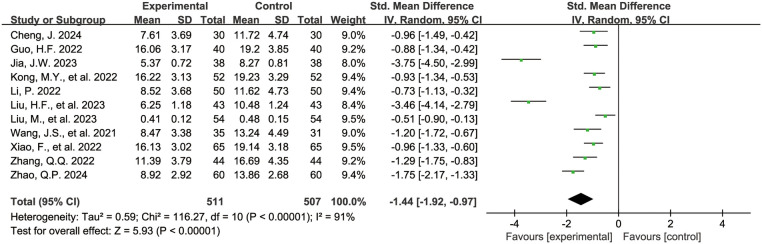
Forest plot of C-reactive protein levels at the end of treatment.

The Figure 6 shows the forest plot of C-reactive protein levels at the end of treatment. 11 studies (16, 19, 21–23, 25, 26, 36, 38, 42, 43) reported C-reactive protein levels, involving 1,018 children, with 511 in the experimental group and 507 in the control group. Compared with the control group, children treated with acetylcysteine combined with budesonide had lower C-reactive protein levels, and the differences were statistically significant (SMD = −1.44, 95% CI = −1.92 to −0.97, *I*^2^ = 91%).

### 3.6 Sensitivity analyses

We conducted sensitivity analyses for the results of clinical overall response rate and the total incidence of adverse events to examine their stability. After excluding studies one by one, the pooled results for clinical overall response rate (Supplementary Figure S3A) and overall incidence of adverse events (Supplementary Figure S3B) showed no significant changes, indicating that the conclusions of this meta-analysis are robust.

## 4 Discussion

This meta-analysis, based on 29 randomized controlled trials involving 4,300 children, demonstrated that acetylcysteine combined with budesonide achieved a significantly higher overall clinical efficacy rate for the treatment of Mycoplasma pneumonia in children compared to the comparison group. Additionally, no significant difference was observed in the incidence of adverse events between the two groups. These findings suggest that acetylcysteine combined with budesonide is a viable treatment option.

The following mechanisms may explain the synergistic effects of acetylcysteine combined with budesonide: (1) Synergistic anti-inflammatory effects. Budesonide, a glucocorticoid, alleviates airway inflammation by inhibiting the activation and proliferation of inflammatory cells, such as macrophages and lymphocytes, and reducing the release of inflammatory mediators like interleukin-6 and tumor necrosis factor-α (TNF-α) (44, 45). It also reduces the synthesis of inflammatory mediators by inhibiting transcription factors such as nuclear factor-κB (NF-κB), decreases vascular permeability to reduce airway edema, and improves ventilation (46, 47). Meanwhile, acetylcysteine scavenges reactive oxygen species via its antioxidant properties, reduces oxidative stress-induced lung tissue damage, and inhibits the NLRP3 inflammasome pathway to further decrease inflammatory mediators (48). Together, these agents synergistically suppress inflammatory responses and mitigate airway damage through complementary mechanisms, facilitating subsequent treatment. (2) Synergistic mucolytic effects. Acetylcysteine reduces sputum viscosity by disrupting disulfide bonds in glycoprotein peptide chains through its thiol groups (49). It also enhances ciliary movement in the airway and increases pulmonary surfactant secretion, which facilitates sputum clearance and improves gas exchange (50, 51). Budesonide complements this action by reducing airway inflammation and mucus secretion, creating an optimal environment for the mucolytic effects of acetylcysteine. Additionally, budesonide alleviates airway spasm and improves ventilation, enabling acetylcysteine to better reach affected areas and dissolve sputum. These synergistic effects significantly improve sputum clearance and reduce airway obstruction. (3) Synergistic immunomodulatory effects. Acetylcysteine regulates immune function by inhibiting the NLRP3 inflammasome pathway, reducing the release of inflammatory mediators, and enhancing the body's antioxidant capacity, thereby facilitating pathogen clearance (52). Furthermore, budesonide suppresses the activation and proliferation of inflammatory cells, reduces the release of inflammatory mediators, and restores immune system balance, preventing tissue damage caused by excessive immune responses (53, 54). Together, these agents effectively control inflammation and promote recovery through complementary immunomodulatory mechanisms. (4) Synergistic effects on the alleviation of clinical symptoms. Acetylcysteine alleviates cough by reducing sputum viscosity and facilitating sputum clearance, while budesonide reduces airway inflammation and relieves airway spasm, improving dyspnea. Moreover, acetylcysteine enhances ciliary movement and increases pulmonary surfactant secretion, contributing to improved pulmonary ventilation. Budesonide alleviates airway hyperresponsiveness and inflammation, promoting the absorption of pulmonary inflammation and tissue repair. These synergistic effects markedly enhance the alleviation of clinical symptoms.

Notably, in the subgroup analyses of two primary outcomes (overall clinical efficacy rate and total incidence of adverse events), the low-risk group exhibited lower heterogeneity among studies compared to the high-risk group, indicating that elevated bias risk in the included studies (primarily arising from the majority of studies being rated as “unclear” or “high risk” in terms of blinding and allocation concealment) may be a significant contributor to the heterogeneity observed in this meta-analysis.

In this meta-analysis, the remission times for cough, pulmonary rales, and fever, as well as the extent of reduction in C-reactive protein (CRP) levels, demonstrated substantial heterogeneity (*I*^2^ = 97%, 97%, 95%, and 91%, respectively). This pronounced heterogeneity may arise from several factors: (1) Variability in study populations, including heterogeneous age distributions among children and differences in baseline health status, such as the presence of comorbidities, which may influence treatment response; (2) Variations in intervention protocols, with discrepancies in the dosage, administration frequency, and nebulization devices or techniques used for acetylcysteine and budesonide, thereby affecting drug delivery and therapeutic efficacy in the respiratory tract; (3) Differences in disease severity, as the included studies lacked standardized classification or stratification of Mycoplasma pneumoniae pneumonia severity, leading to inconsistent disease severity among children across studies, which in turn impacted treatment outcomes and symptom resolution times; (4) Inconsistencies in outcome assessment criteria, where subjective differences in evaluators' interpretation of clinical efficacy may have occurred, and variations in detection methods, reagents, and reference ranges for CRP measurement may have contributed to result discrepancies. The interplay of these factors likely accounts for the substantial heterogeneity observed in certain outcome measures in this study.

This meta-analysis summarized the latest comprehensive evidence regarding the use of acetylcysteine combined with budesonide in the treatment of pediatric Mycoplasma pneumonia, offering novel insights for the clinical management of this condition.

However, this study also presents certain limitations. First, the methodological quality of the included studies varies. Some studies provided insufficient details on randomization processes and blinding, which could lead to subjective bias and affect the objectivity and accuracy of the results. Secondly, the age group of participants is limited. According to The International Council for Harmonisation of Technical Requirements for Pharmaceuticals for Human Use (ICH) E11(R1) guidelines, children are defined as aged 2–11 years (55), whereas the included children primarily fall within the age range of 2–7 years. This may not fully reflect responses to combined treatment across all pediatric age groups, limiting the generalizability of the study's conclusions. Additionally, the severity of illness was not classified. Children with different pneumonia severities may respond differently to treatment. The absence of stratified analysis may affect the accurate assessment of treatment outcomes, making it challenging to determine the specific benefits of combined therapy in mild, moderate, or severe cases. Third, all included studies were conducted at a single center, which could introduce selection bias. Variations in healthcare systems, environmental conditions, and population health across regions may affect the external validity of the results. Multi-center studies could reduce this bias and enhance the applicability of findings. Fourth, some studies employed relatively simple statistical methods. For complex data affected by multiple factors, more advanced techniques such as multivariate analysis were not used to control for confounding factors, potentially impacting the accuracy and reliability of the results. Furthermore, in the adverse event analysis conducted in this study, we observed that the combined treatment with acetylcysteine and budesonide may reduce the incidence of diarrhea. Nevertheless, it is imperative to acknowledge the limitations inherent in this finding. First, this observation relies on a limited number of studies, each characterized by relatively small sample sizes, which may result in inadequate statistical power and consequently compromise the robustness of the conclusions. Moreover, the analysis of these adverse events revealed wide confidence intervals, suggesting that the current research may not accurately capture the true differences in the incidence rates of these events. Accordingly, this conclusion warrants cautious interpretation.

In summary, our results demonstrate that the combination of Acetylcysteine and Budesonide demonstrates superiority in multiple dimensions, including clinical efficacy rate, anti-inflammatory effects, improvement of clinical symptoms, and reduction of adverse events. This multi-dimensional therapeutic effect contributes to a more comprehensive intervention in the complex pathological process of pediatric Mycoplasma pneumonia.

Although the combination therapy demonstrates potential clinical benefits overall, individualized assessment and treatment based on the specific conditions of the patients are still necessary in clinical practice. Future studies should further explore the differences in treatment responses among different children and develop individualized treatment plans based on factors such as age, disease severity, and underlying conditions, with the aim of maximizing therapeutic efficacy.

## Supplementary material

Supplementary Figure S1 Funnel plot for overall clinical efficacy rate.

Supplementary Figure S2 Funnel plot of the overall incidence of adverse events.

Supplementary Figure S3 Sensitivity analysis of **(A)** clinical overall response rate, **(B)** overall incidence of adverse events.
