# Supplementary figures and images for: The efficacy and safety of acetylcysteine combined with budesonide nebulization in treating mycoplasma pneumonia in children: a meta-analysis
Source: Front Pediatr. 2025 Jun 20;13:1574257. doi: 10.3389/fped.2025.1574257 (PMC12226471; doi:10.3389/fped.2025.1574257)

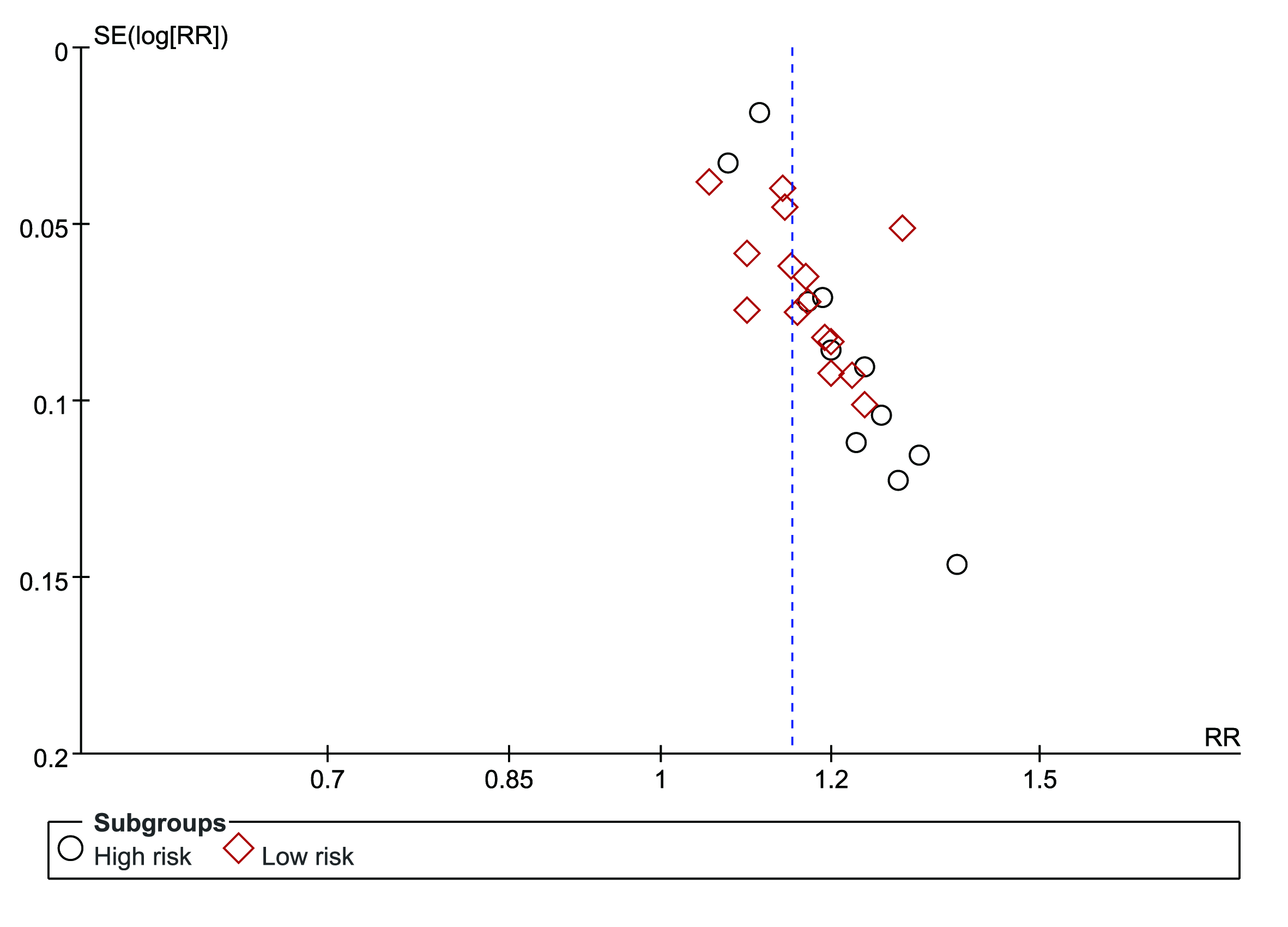

Supplement: Supplementary Figure S1 — Funnel plot for overall clinical efficacy rate. [file Image1.tif]

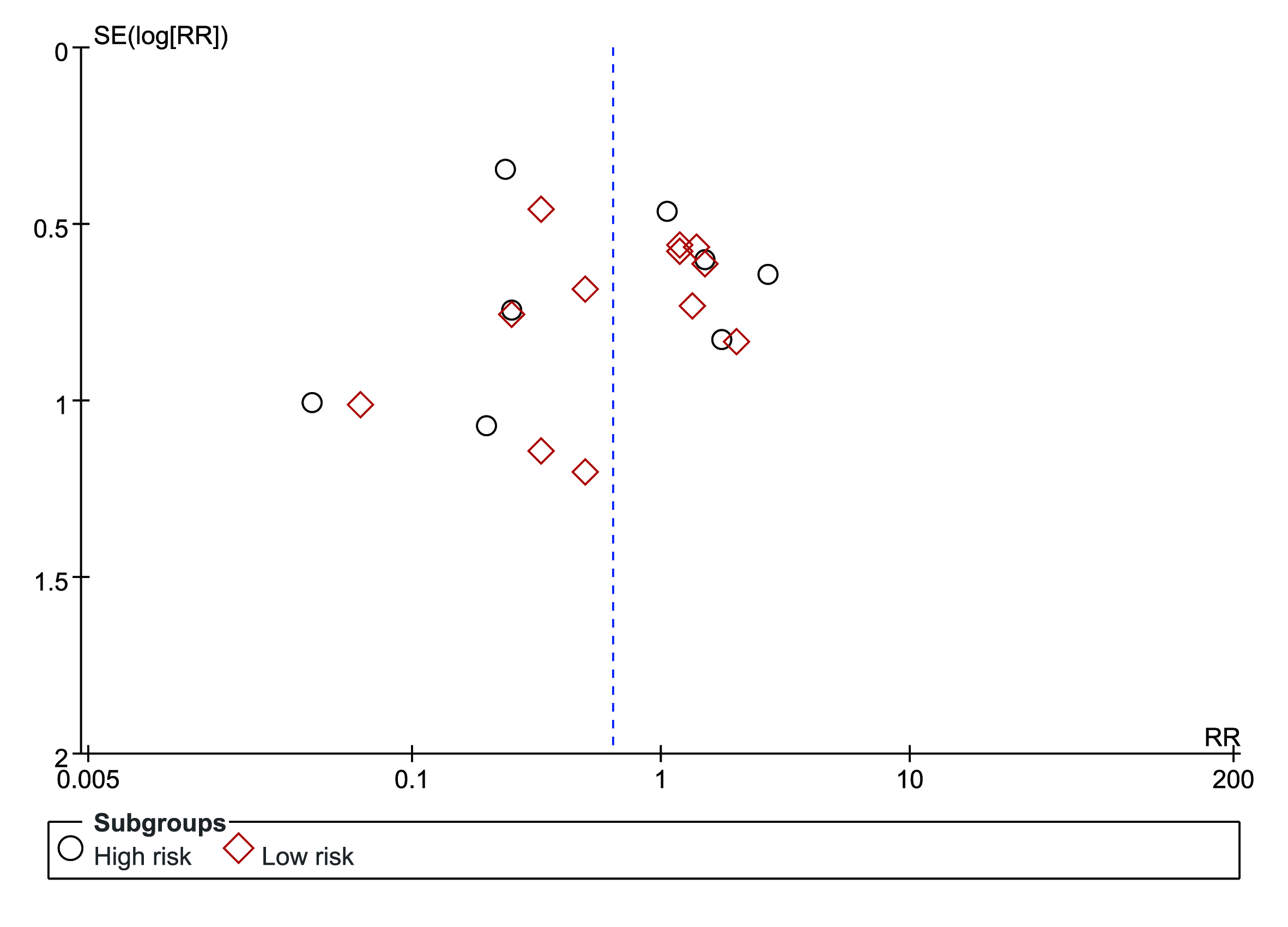

Supplement: Supplementary Figure S2 — Funnel plot of the overall incidence of adverse events. [file Image2.tif]

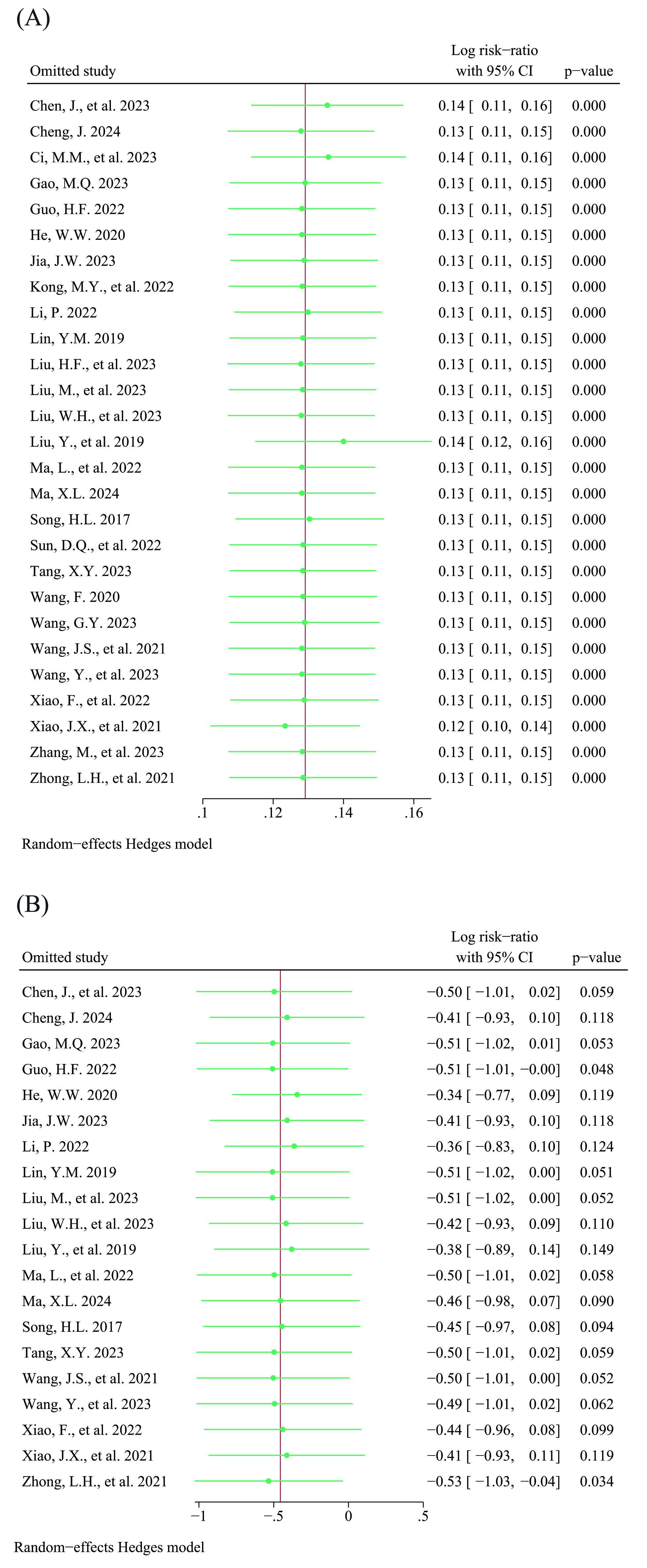

Supplement: Supplementary Figure S3 — Sensitivity analysis of (A) clinical overall response rate, (B) overall incidence of adverse events. [file Image3.tif]
